# Associations of Clinical Characteristics and Etiology With Death in Hospitalized Chinese Children After Spontaneous Intracerebral Hemorrhage: A Single-Center, Retrospective Cohort Study

**DOI:** 10.3389/fped.2020.576077

**Published:** 2021-02-04

**Authors:** Xiaoyan Huang, Zicheng Cheng, Ye Xu, Lingfan Xia, Zhenxiang Zhan, Tong Xu, Yungang Cao, Zhao Han

**Affiliations:** Department of Neurology, The Second Affiliated Hospital and Yuying Children's Hospital of Wenzhou Medical University, Wenzhou, China

**Keywords:** children, spontaneous ICH, cerebrovascular disease, arteriovenous malformation, low GCS, herniation syndrome

## Abstract

**Objective:** We retrospectively analyzed clinical characteristics, etiology, and mortality risk factors in pediatric cases of non-traumatic spontaneous intracerebral hemorrhage.

**Methods:** This study involved children between 29 days and 18 years old with confirmed spontaneous intracerebral hemorrhage based on head CT or MRI at the Second Affiliated Hospital of Wenzhou Medical University and Yuying Children's Hospital from January 2008 to March 2020. Demographic and clinical characteristics, etiology, imaging, and treatment data were collected at baseline. Potential risk factors of in-hospital death were identified using univariate analysis and multivariate logistic regression.

**Result:** A total of 200 children (126 males, median age 5 years) were included in the study. Clinical symptoms of spontaneous intracerebral hemorrhage were typically non-specific (79.5%). One third of patients (31.1%) had a Glasgow Coma Scale score (GCS) ≤ 8, and nearly two-thirds (60.5%) showed a combination of ventricular hemorrhage or subarachnoid hemorrhage. Supratentorial hemorrhage was more common. Cerebrovascular disease (37.0%) and hematological disease (33.5%) were the most frequent etiologies of spontaneous intracerebral hemorrhage. Most patients (74.5%) received non-surgical treatment, while 25.5% received surgical treatment. After an average of 12 days of treatment, 167 children (83.5%) survived and 33 (16.5%) died. Multivariate logistic regression showed herniation syndrome, and low GCS (≤ 8) to be associated with increased risk of mortality, while hemorrhage due to arteriovenous malformation was associated with lower risk of mortality.

**Conclusion:** Our data suggest that cerebrovascular disease is the most common cause of spontaneous intracerebral hemorrhage among children, and that arteriovenous malformation is associated with lower risk of death in hospital. Conversely, the presence of herniation syndrome, low GCS (≤ 8) increase risk of in-hospital mortality. Our results underscore the importance of timely imaging and supplementary examinations in cases of suspected spontaneous intracerebral hemorrhage.

## Introduction

Stroke is a major cause of acquired brain injury and morbidity in children (1). Pediatric spontaneous intracerebral hemorrhage (sICH) is non-traumatic brain parenchymal bleeding with or without intraventricular extension in patients between 29 days and 18 years old (2). It is a common cause of acute brain injury in children, and it leads to significant mortality and morbidity. The absolute number of childhood strokes increased by 1.5 times from 1990 to 2013 globally, with hemorrhagic strokes accounting for about half of pediatric strokes (3–5). Detection of stroke in children has improved with the advancement of neuroimaging technology, but survivors often suffer long-term neurological, cognitive and adaptive behavioral damage (1).

The clinical and radiological characteristics and risk factors of sICH in children are different from those in adults (6), which means that different parameters must be considered for accurate diagnosis. Several studies have examined sICH caused by arteriovenous malformations (AVMs), aneurysms, cavernoma, and brain tumors in children, but few clinical studies have investigated the etiology, clinical characteristics and outcomes of sICH in children (2, 7).

Therefore, we performed a retrospective analysis of children diagnosed with sICH. The results of this analysis could help define diagnostic criteria and guide the treatment of children with sICH.

## Materials and Methods

### Study Design and Patients

This study complied with our hospital's scientific principles and research ethics standards. Due to its retrospective nature, our institutional review board waived the requirement for written informed consent. The data used for analysis are listed in the tables of this article and were approved by the Second Affiliated Hospital of Wenzhou Medical University.

The participants in this study were recruited from the Yuying Children's Hospital of Wenzhou Medical University from January 2008 to March 2020. Participants were children between 29 days and 18 years old who experienced pediatric sICH, as defined in World Health Organization standards (8), with or without intraventricular hemorrhage. Additionally, head rapid computed tomography scanning (CT) or magnetic resonance imaging (MRI) must have been used to distinguish sICH from ischemic stroke. Causes of hemorrhage were determined using MRI, magnetic resonance angiography (MRA), computed tomography angiography (CTA) or digital subtraction angiography (DSA); causes could include AVMs, tumors, MoyaMoya disease, aneurysm and cerebral cavernous malformations (2). Exclusion criteria were: history of brain trauma, bleeding limited to the intraventricular or subarachnoid epidural, or diagnosis with venous embolism or arterial ischemic stroke. Cases were identified from a hospital discharge database based on their “International Classification of Disease, 10th revision” (ICD-10-CM) code: primary diagnosis code 85 was used to identify children admitted with sICH.

### Data Collection

#### Demographics and Clinical Parameters

Demographic characteristics included sex, age and length of hospital stay (in days). Clinical characteristics consisted of blood pressure, body temperature, symptoms, laboratory tests [leukocyte, lymphocyte, hemoglobin, international normalized ratio (INR), activated partial thromboplastin time (APTT), prothrombin time (PT)], presenting symptoms, treatment and days in the intensive care unit (ICU).

We classified hypertension according to the 95th percentile method from the standard blood pressure table for children published by the fourth report of the National Heart, Lung, and Blood Institute (9). Blood pressure tables included the 50th, 90th, 95th, and 99th percentiles (with standard deviations) by sex, age, and height. Blood pressure was classified as normal if it was below the 90th percentile; pre-hypertension, if it was between the 90th and 95th percentiles, except in adolescents, in whom pre-hypertension was defined as ≥120/80 mmHg; stage I hypertension, if it was between the 95th and 99th percentile plus 5 mmHg; or stage II hypertension, if it was in the >99th percentile plus 5 mmHg. Stage II hypertension accompanied by clinical symptoms was diagnosed as acute hypertension (10).

Presenting symptoms were classified as non-specific [headache, vomiting, convulsions, Glasgow Coma Scale score (GCS), or Children Coma Scale on admission], or focal deficit (hemiplegia, facial paralysis, dysarthria, aphasia, ataxia, sensory and eye movement).

#### Imaging Analysis

The following information was extracted from CT or MRI: location of cerebral hemorrhage (lobe, deep brain, brainstem and cerebellum), presence of subarachnoid hemorrhage or intraventricular hemorrhage (IVH), presence of herniation syndrome or hydrocephalus, supratentorial or infratentorial (cerebellum and brain stem), and hematoma volume. In addition, cerebrovascular malformations were assessed using MRA, CTA, or DSA. Hematoma volume was calculated using the equation ABC/2, where A = the greatest hemorrhage diameter by CT, B = the diameter at 90 degrees to A, and C = the approximate number of CT slices with hemorrhage multiplied by the slice thickness (11).

#### Etiology Classification

We classified sICH cases by etiology based on a recent review (2). The following etiologies were used: cerebrovascular disease, hematologic system disease, infection, tumor, other systemic disease (hypertensive encephalopathy, multiple organ dysfunction syndrome), or unknown cause.

### Statistical Analysis

Statistical analysis was performed using SPSS 22.0 (IBM, Chicago, IL, USA). Demographic continuous variables were reported as median and range, and they were analyzed by ANOVA or Mann–Whitney *U*-test. Categorical variables were reported in numbers and percentages, and group differences were assessed by chi-squared test or Fisher exact test. Continuous data were compared using paired and unpaired *t*-tests as appropriate. Binary logistic regression was used to determine possible predictors of death in children with sICH. Differences associated with a two-sided *P* < 0.05 were considered statistically significant. Results were expressed as odds ratio (OR) and 95% confidence interval (CI).

## Results

### Demographic and Clinical Characteristics of Children With sICH

We collected information on a total of 200 patients with childhood sICH. Over half were males (*n* = 126) and the median age was 5 years old. Nearly half the children (45.0%) were in infancy (29 days - 1 year), and only 4.0% were in adolescence (15–18 years). Median length of hospital stay was 12 (4 −19.5) days. The median systolic blood pressure was 104 (90–115) mmHg, and the median diastolic blood pressure was 63 (52–72) mmHg. The median body temperature was 37.1 (36.8–37.6)°C. The following median laboratory values were determined: leukocytes, 12.8 (9.4–18.4) × 10^9^/L; lymphocytes, 2.6 (1.5–4.5) × 10^9^/L; INR, 1.1 (1.0–1.2); and hemoglobin, 117 (90–129) g/L; APTT, 38.6 (34.2–46.6) s; and PT, 15.5 (13.3–15.5) s. The first symptoms of hemorrhagic stroke were mostly non-specific: vomiting (60.0%), headache (40.0%), and convulsion (34.5%). Consciousness was evaluated using the GCS: 55.6% of children had a GCS of 12–15, while 31.1% had GCS of 3–8. Only 20.5% of children had symptoms of focal neurological defects.

### CT or MRI Findings in Children With sICH

**Figure 1:**
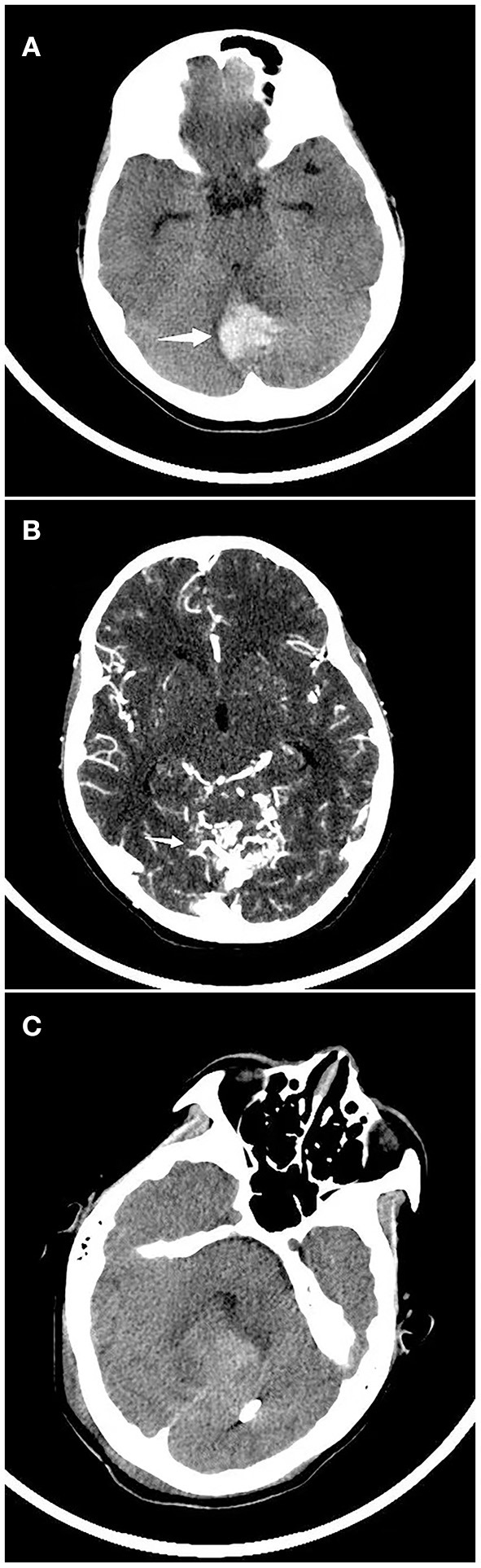
Continuous CT imaging of a child with spontaneous cerebral hemorrhage. **(A)** A child was admitted to the hospital due to vomiting and dizziness, and a head CT examination showed that that cerebellar hemorrhage. **(B)** CT angiography suggested arteriovenous malformations. **(C)** Head CT was reviewed 2 weeks after arteriovenous malformation embolization.

The volume of cerebral hematoma was calculated from head CT or MRI examination (Table 1); the median hematoma volume was 14.8 (5.1–40.0) ml. Most diagnoses were sICH with intraventricular or subarachnoid hemorrhage (121 cases, 60.5%), whereas 79 cases (39.5%) were diagnosed as isolated ICH. The sICH affected most often the supratentorial region (84.5%). We found that 96 children (48.2%) had cerebral hemorrhage on the left, 71 (35.7%) on the right, and 32 (16.1%) on both sides. Specifically, 77.0% of sICH cases occurred in a lobe of the brain, 15.5% in the deep brain (internal capsule, basal ganglia, thalamus), 14.0% in the cerebellum (Figure 1), and 3.0% in the brain stem. Of the patients, 18.5% developed herniation syndrome, and 65.5% were admitted to the ICU. In univariate analysis, brain herniation (*p* = 0.000), infratentorial ICH (*p* = 0.041), sides (*p* = 0.000) and isolated ICH (*p* = 0.019) related to sICH in children.

**Table 1:** Demographic, clinical, and imaging characteristics of children with sICH at hospital admission (*n* = 200).

**Characteristic**	**Value**
Male	126 (63.0)
Age, year	5 (0.24–10)
Length of hospital stay, days	12 (4–19.5)
Systolic blood pressure, mmHg	104 (90–115)
Diastolic blood pressure, mmHg	63 (52–72)
Body temperature, °C	37.1 (36.8–37.6)
Non-typical	
Vomit	120 (60.0)
Headache	80 (40)
Convulsions	69 (34.5)
Focal deficits	41 (20.5)
GCS on admission	
Mild (12–15)	109 (55.6)
Moderate (9–11)	26 (13.3)
Severe (3–8)	61 (31.1)
Laboratory analysis	
Leukocytes, *10∧9/L	12.8 (9.4–18.4)
Lymphocytes, *10∧9/L	2.6 (1.5–4.5)
Hemoglobin, g/L	117 (90–129)
INR	1.1 (1.0–1.2)
APTT, s	38.6 (34.2–46.6)
PT, s	15.5 (13.3–15.5)
Hemorrhage characteristics	
Isolated ICH	79 (39.5)
ICH with subarachnoid or IVH	121 (60.5)
Hydrocephalus	16 (8.0)
Herniation syndrome	37 (18.5)
Side affected by the lesion	
Left	96 (48.2)
Right	71 (35.7)
Bilateral	32 (16.1)
Supratentorial ICH	169 (84.5)
Infratentorial ICH	31 (15.5)
Localization of lesion	
Lobe	154 (77.0)
Deep brain	31 (15.5)
Cerebellum	28 (14.0)
Brainstem	6 (3.0)
Hematoma volume, mL	14.8 (5.1–40.0)
Treatment	
Dehydration	179 (89.5)
Tracheal intubation	47 (23.5)
Packed red cell transfusion	56 (28.0)
Platelet transfusion	14 (7.0)
FFP transfusion	69 (34.5)
Surgery	51 (25.5)
Decompressive craniectomy	12 (6.0)
Hematoma removal	31 (15.5)
Ventricular drainage	14 (7.0)
AVM resection	18 (9.0)
In the ICU	131 (65.5)

*Values are n (%) or mean (range)*.

*Abbreviations: sICH, spontaneous intracerebral hemorrhage; ICH, intracerebral hemorrhage; GCS, Glasgow Coma Scale; INR, international normalized ratio; APTT, activated partial thromboplastin time; PT, prothrombin time; IVH, intraventricular hemorrhage; FFP, fresh frozen plasma; AVM, arteriovenous malformation; ICU, intensive care unit*.

### Treatment

All children received medical treatment to control the increase in intracranial pressure (ICP) while in hospital: 89.5% of children were subjected to dehydration, while 25.5% underwent surgery, such as decompressive craniectomy, hematoma removal, ventricular drainage, and AVM resection. During treatment, 47 children underwent endotracheal intubation, 56 received transfusions of packed red cells, 14 received transfusions of platelets, and 70 received transfusions of fresh frozen plasma. In the multivariate analysis, there were no significant difference in treatment.

### Etiology of sICH

The most frequent causes of sICH were cerebrovascular disease (37.0%), followed closely by hematologic system disease (33.5%) (Table 2). Cerebrovascular diseases were most common among children aged 7–14 years. Specific diseases that contributed most often to sICH were AVM (28.5%) (Figure 1), cavernoma (2.0%), aneurysm (2.5%), and MoyaMoya disease (1.5%). In contrast, hematologic diseases were most likely to be present in infants and young children (aged 29 days to 3 years); these included vitamin K deficiency (20.0%), hemophilia (5.0%), leukemia (4.5%), thrombocytopenia (3.5%), and aplastic anemia (0.5%). None of the children with vitamin K deficiency in our cohort received anticoagulant drugs. Due to the technical limitations of the imaging systems, the cause of 44 children (21.9%) could not be clearly determined, and their coagulation function and blood smear examination were normal, whose sICH was therefore attributed to unknown causes. Small proportions of cases were attributed to tumors (3.5%), infections (1.5%) or other causes such as hypertensive encephalopathy or multiple organ dysfunction syndrome (2.5%). Among the tumor patients, 5 were supratentorial space-occupying with bleeding, and 2 were infratentorial. In terms of classification, 1 was medulloblastoma, 1 was melanoma, and the remaining 5 were transferred to a higher-level hospital without pathological biopsy. Among the 3 infected patients, one was with purulent encephalitis, one was with viral encephalitis, and the other was with parasite infection. They all developed intracranial hemorrhage after infection.

**Table 2:** Etiology of sICH in different age groups of children (*n* = 200).

**Etiology**	**Total**	**29 days-3 years**	**4–6 years**	**7–10 years**	**11–14 years**	**15–18 years**
Vascular	74 (37.0)	7 (3.5)	9 (4.5)	30 (15.0)	23 (11.5)	6 (3.0)
AVM	57 (28.5)	1 (0.5)	5 (2.5)	28 (14.0)	19 (9.5)	4 (2.0)
Aneurysm	5 (2.5)	2 (1.0)	2 (1.0)	1 (0.5)	0	0
Cavernoma	4 (2.0)	1 (0.5)	0	0	1 (0.5)	2 (1.0)
MoyaMoya	3 (1.5)	0	0	1(0.5)	2 (1.0)	0
Other vascular	5 (2.5%)	3 (1.5)	1 (0.5)	0	1 (0.5)	0
Hematologic	67 (33.5)	56 (28.0)	4 (2.0)	4 (2.0)	3 (1.5)	0
Hemophilia A or B	10 (5.0)	8 (4.0)	2 (1.0)	0	0	0
Vitamin K deficiency	40 (20.0)	40 (20.0)	0	0	0	0
Leukemia	9 (4.5)	3 (1.5)	1 (0.5)	4 (2.0)	1 (0.5)	0
Thrombocytopenia	7 (3.5)	5 (2.5)	1 (0.5)	0	1 (0.5)	0
Aplastic anemia	1 (0.5)	0	0	0	1 (0.5)	0
Infection	3 (1.5)	1 (0.5)	0	1 (0.5)	1 (0.5)	0
Tumor	7 (3.5)	5 (2.5)	1 (0.5)	1 (0.5)	0	0
Unknown	44 (22.0)	19 (9.5)	6 (3.0)	11 (5.5)	6 (3.0)	2 (1.0)
Other^a^	5 (2.5)	2 (1.0)	0	0	3 (1.5)	0

*Values are n (%)*.

a *Multiple organ dysfunction syndrome or hypertension*.

*sICH, spontaneous intracerebral hemorrhage; AVM, arteriovenous malformation*.

### Risk Factors for In-Hospital Death in Children With sICH

After treatment, the health of 167 children improved and they were discharged, while 33 children did not survive according to hospital records. Univariate analysis identified several variables that were significantly associated with death in hospital (Table 3): age, AVM, leukemia, low GCS (3–8), leukocytes, lymphocytes, PT, isolated ICH, lesion location on the side of the brain, herniation syndrome, admission to the ICU, infratentorial ICH, tracheal intubation, transfusion with packed red cells or platelets. Factors associated with mortality in multivariate analysis included herniation syndrome (OR 21.951, 95% CI 3.830–125.805), AVM (OR 0.965, 95% CI 0.819–1.137), and low GCS (OR 15.415, 95% CI 2.804–84.747) (Table 4).

**Table 3:** Screening for risk factors of in-hospital death among children after sICH (*n* = 200).

**Factor**	**Multivariate analysis**		
	**OR**	**95% CI**	***P*-value**
Age	0.965	0.819–1.137	0.674
AVM	0.034	0.002–0.464	0.011^*^
Leukemia	0.874	0.006–128.729	0.958
Convulsions	3.295	0.696–15.608	0.133
Focal deficits	0.146	0.015–1.382	0.093
Low GCS	15.415	2.804–84.747	0.002^*^
Leukocytes, *10∧9/L	1.004	0.991–1.017	0.592
Lymphocytes, *10∧9/L	1.022	0.974–1.073	0.367
PT, s	0.981	0.959–1.004	0.107
Herniation syndrome	21.951	3.830–125.805	0.001^*^
Infratentorial	2.175	0.301–15.735	0.442
Isolated ICH	4.184	0.659–26.546	0.129
Sides			0.199
left	0.184	0.029–1.166	0.072
right	0.306	0.042–2.237	0.243
Tracheal intubation	2.426	0.521–11.288	0.259
Packed red cell transfusion	0.818	0.134–4.982	0.827
Platelet transfusion	1.689	0.070–40.956	0.747
In the ICU	0.974	0.102–9.264	0.981

*sICH, spontaneous intracerebral hemorrhage; OR, odds ratio; CI, confidence interval; AVM, arteriovenous malformation; GCS, Glasgow Coma Scale; PT, prothrombin time; ICH, intracerebral hemorrhage; ICU, intensive care unit*.

* *p <0.05*.

**Table 4:** Univariate analysis of risk factors associated with in-hospital death in children after sICH (*n* = 200).

**Characteristic**	**Survive** (*n* **= 167)**	**Die** (*n* **= 33)**	***P*-value**
Male	107 (64.1)	19 (57.6)	0.507
Age	6 (0.23–10)	2 (0.26–8.5)	0.079
AVM	55 (32.9)	2 (6.1)	0.002^*^
Leukemia	3 (1.8)	6 (18.2)	0.001^*^
Tumor	4 (2.4)	3 (9.1)	0.089
Unknown	35 (21.0)	9 (21.7)	0.413
Non-typical			
Vomit	100 (59.9)	20 (60.6)	0.958
Headache	70 (41.9)	9 (27.3)	0.108
Convulsions	53 (31.7)	16 (48.5)	0.061
Focal deficits	39 (23.4)	3 (9.1)	0.068
Low GCS	34 (20.4)	27 (81.8)	0.000^*^
Laboratory			
Leukocytes, *10∧9/L	12.1 (9.1–17.1)	17.7 (12.5–25.8)	0.000^*^
Lymphocytes, *10∧9/L	2.4 (1.5–4.4)	3.6 (2.4–7.7)	0.010^*^
APTT, s	38.1 (34.1–46.0)	40.8 (34.6–55.1)	0.278
PT, s	14.1 (13.3–14.9)	14.9 (13.9–23.6)	0.008^*^
Hemorrhage characteristics			
Isolated ICH	72 (42.9)	7 (21.2)	0.019^*^
Hydrocephalus	14 (8.3)	2 (6.1)	1.000
Herniation syndrome	19 (11.4)	18 (54.5)	0.000^*^
Sides			
Left	86 (51.4)	10 (30.3)	0.000^*^
Right	62 (37.1)	9 (27.3)	
Bilateral	19 (11.4)	13 (39.4)	
Infratentorial ICH	22 (13.2)	9 (27.3)	0.041^*^
Hematoma volume	13.7 (3.75–39.9)	20.8 (7.5–43.7)	0.263
Treatment			
Dehydration	149 (89.2)	30 (90.9)	1.000
Tracheal intubation	26 (15.6)	21 (63.6)	0.000^*^
Packed red cell transfusion	42 (25.1)	14 (42.4)	0.043^*^
Platelet transfusion	7 (4.1)	7 (21.2)	0.003^*^
FFP transfusion	54 (32.3)	15 (45.5)	0.147
Surgery	46 (27.5)	5 (15.2)	0.136
Decompressive craniectomy	9 (5.4)	3 (9.1)	0.422
Hematoma removal	28 (16.8)	3 (9.1)	0.266
Ventricular drainage	11 (6.6)	3 (9.1)	0.707
AVM resection	17 (10.2)	1 (3.0)	0.318
In the ICU	100 (59.9)	31 (93.9)	0.000^*^

*Values are n (%) or mean (range)*.

*sICH, spontaneous intracerebral hemorrhage; AVM, arteriovenous malformation; GCS, Glasgow Coma Scale; APTT, activated partial thromboplastin time; PT, prothrombin time; FFP, fresh frozen plasma; ICH, intracerebral hemorrhage; ICU, intensive care unit*.

* *p < 0.05*.

## Discussion

Spontaneous ICH remains an important cause of morbidity and mortality worldwide (12). The present study provides detailed insights into clinical characteristics, etiology, and risk factors for death among children hospitalized for sICH. According to the data released by the Global Burden of Disease (GBD), stroke prevalence among children increased significantly worldwide from 1990 to 2013. Moreover, the mortality rate of infants under 1 year old in 2013 was 6.1 (95% UI 5.0–7.5) per 100,000. In 2013, the prevalence of ischemic stroke was similar to that of hemorrhagic stroke, but the latter was associated with a 6- to 7-fold higher mortality rate (3).

The patients included in our study were younger and mostly male (male: female =1.7:1) and male predominance was also observed in a previous study of sICH in children (13). For reasons that remain unclear, males show higher mortality and daily disease burden after stroke than females (3, 5). Interestingly, many clinical parameters of our patients with sICH were normal, including body temperature and blood pressure. In our study, 41.0% of children had a body temperature higher than 37.3°C. Fever is quite common after sICH, especially in patients with intraventricular hemorrhage, and it may exacerbate brain damage (14, 15). In contrast to our findings in children, blood pressure is elevated in many adults after sICH (16, 17). In fact, the protective mechanism in raised ICP to maintain cerebral perfusion due to autoregulation, is increased systolic blood pressure. However, the children in our study did not show a significant increase in systolic blood pressure, which may render them more vulnerable to shock. The children with sICH in our study who died in hospital showed significantly higher counts of white blood cells and lymphocytes than children with sICH who survived. De-margination of the central leucocyte pool likely causes leukocytosis following hemorrhage.

Clinical symptoms of hemorrhagic stroke in children are different from those of ischemic stroke, so suspected cases of sICH should be examined in detail. Indeed, our analysis of 200 children with sICH showed the most frequent presenting signs to be non-specific, such as vomiting, headache and altered consciousness. Among children who died in the hospital, 81.8% had GCS ≤ 8, and multivariate analysis identified GCS ≤ 8 as an independent risk factor for death. The non-specific manifestations probably reflect a combination of sudden onset of certain clinical symptoms, followed by progressive deterioration of the nervous system (18). Therefore, it is impossible to know whether focal neurological symptoms are reflective of the ischemia or bleeding based on clinical symptoms alone. In order to identify children with sICH as soon as possible, we require a head imaging examination. CT of the head is the preferred method for diagnosing sICH because it is accessible to most medical centers and provides rapid results. Especially in the case of children showing altered consciousness in the acute phase, this translates to quicker decisions by clinicians about the cause of the sICH and course of treatment, which improves prognosis and can reduce risk of sICH recurrence (2, 19). The prevalence of many CT or MRI findings was significantly different between our patients who died in-hospital and those who survived. In fact, herniation syndrome proved to be an independent risk factor of in-hospital death. The high incidence of early neurological deterioration after sICH is associated with symptoms of active bleeding that may last for several hours, so early identification and diagnosis are particularly important.

In our study, all children with sICH received medical treatment. 25.5% of cases received surgical, which involved such procedures as decompressive craniectomy, hematoma removal or ventricular drainage. Surgical treatment was not a predictor of in-hospital death. Studies suggest that most patients with spontaneous supra-cerebral hemorrhage do not benefit from early surgery (20), while the surgery also does not appear to increase risk of mortality or disability at 6 months. For patients with spontaneous superficial intracerebral hemorrhage (10–100 ml) and no intraventricular hemorrhage, the survival advantage of early surgery appears to be small but clinically significant (21). Dehydration was often used to treat acute elevated ICP, and our cohort received transfusions of red cells, platelets, and fresh frozen plasma, reflecting the high prevalence of vitamin K deficiency. If there is loss of respiratory drive, loss of protective airway reflexes, or cardiorespiratory instability, the patient should be intubated and mechanically ventilated (18). Patients with sICH and brain herniation often develop apnea or acute respiratory distress syndrome, which necessitates tracheal intubation.

Unlike adults who tend to have more cases of primary (hypertension) ICH, children tend to suffer “secondary ICH” due to vascular malformations or to genetic or acquired coagulopathy (22). Only one child in our study cohort developed sICH associated with hypertension, whereas the most common cause of sICH was AVM. This was consistent with other literature claiming that AVMs often occur in school-age and pre-adolescent children, and that the associated sICH occurs more often in children than adults (4, 13, 23). In sICH associated with AVM, surgical treatment can reduce the risk of rebleeding (24, 25). The second largest proportion of patients in our study, especially infants and young children, suffered from hematological diseases. Vitamin K deficiency was the main cause of these diseases associated with sICH. This reflects a common lack of vitamin K in newborns in Asia (1), given that none of the children in our cohort received anticoagulant drugs. We identified 10 children with hemophilia A or B, corresponding to a prevalence of 5.0% among our sample of children with sICH. Little is known about the association of hemophilia with intracranial hemorrhage in Chinese. It is estimated that 5–10% of severe hemophiliacs of all ages suffer at least one episode of intracranial bleeding during their lifetime (26). The third major etiology of sICH in our patients was “unknown causes,” which highlights the need for a full examination of pediatric patients with suspected sICH. Causes of bleeding in infants, school-aged children, and adolescents are different. Developmental differences associated with structures of the cerebral circulation could explain differences in physiology and hemodynamics of different types of intracranial hemorrhage (23). Interestingly, we found that aneurysms were rarely a cause for sICH, which contrasts with literature that identifies it as a major cause (23). This may reflect that studies have examined older children outside China. In addition, tumors (3.5%), intracranial infection (1.5%), hypertensive encephalopathy (0.5%), and multiple organ dysfunction (2.0%) were uncommon causes of sICH. Only one child in this cohort had secondary hypertensive encephalopathy due to chronic renal insufficiency. The incidence brain neoplasms-related ICH, it is known to be low in adults (<5%), and likely lower in children (2). Present results suggest that hemorrhaging in brain tumors rarely occurs.

Outcomes of hemorrhagic strokes in children vary greatly, with mortality estimated to be between 4 and 54% (27, 28). Children with hemorrhagic stroke often need to be treated in the ICU due to the severity. In the present study, 16.5% of children died in-hospital, 65.5% were admitted to ICU, and 25.5% received surgical treatment. Multivariable analysis identified herniation syndrome and low GCS (3–8) as risk factors of mortality, while AVM as the cause of bleeding was identified to be a protective factor. Poor prognosis has also been associated with increased intracranial hemorrhage volume, changes in mental status within 6 h of treatment, location of hemorrhage, GCS at admission ≤ 7, age < 3 years and hematological disorders (1, 29). Therefore, identifying risk factors for hemorrhagic stroke in children is extremely important for prognosis.

### Strengths and Limitations

Our research was a retrospective analysis of the etiology, clinical features and death-related risk factors of sICH in Chinese children extending over more than 10 years, and it provides some insights for Chinese children with sICH. While our study may help clinicians detect and manage pediatric cases of sICH in the future, our analysis should be treated with caution because it was a single-site study involving a small population. In addition, we were unable to assess whether healthy children are left with disabilities because these data were not routinely collected from sICH patients at our hospital at the time of the study. Future prospective studies are needed to understand changes in causes and prognosis of pediatric sICH, especially with the growing use of vitamin K.

## Conclusions

In this cohort, cerebrovascular disease, especially AVM, was a common cause of sICH in children and was predictive of survival rate. Many patients in our cohort had unspecified symptoms of headache, vomiting and altered consciousness, which suggests that sICH can easily be misdiagnosed. In our study, GCS ≤ 8 and brain herniation were independent risk factors for death. Further research is needed to help predict which pediatric patients are at higher risk of poor prognosis.

## Data Availability Statement

The raw data supporting the conclusions of this article will be made available by the authors, without undue reservation.

## Ethics Statement

The studies involving human participants were reviewed and approved by Department of Neurology, The Second Affiliated Hospital and Yuying Children's Hospital of Wenzhou Medical University, Wenzhou, China. The requirement for written informed consent was waived by the Second Affiliated Hospital of Wenzhou Medical University, due to the retrospective nature of the study.

## Author Contributions

XH, ZH, and ZC contributed to the study conception and design. XH, YX, LX, ZZ, TX, and YC contributed to the data analysis. The first draft of the manuscript was written by XH, and all authors revised it. All authors read and approved the final manuscript.

## Conflict of Interest

The authors declare that the research was conducted in the absence of any commercial or financial relationships that could be construed as a potential conflict of interest.
